# Applications of tetrahedral DNA nanostructures in wound repair and tissue regeneration

**DOI:** 10.1093/burnst/tkac006

**Published:** 2022-03-10

**Authors:** Yikai Dou, Weitong Cui, Xiao Yang, Yunfeng Lin, Xiaohong Ma, Xiaoxiao Cai

**Affiliations:** 1 State Key Laboratory of Oral Diseases, National Clinical Research Center for Oral Diseases, West China Hospital of Stomatology, Sichuan University, Chengdu, 610041, China; 2 Psychiatric Laboratory and Mental Health Center, West China Hospital of Sichuan University, Chengdu, Sichuan, 610064, China; 3 Psychiatric Laboratory and Mental Health Center, the State Key Laboratory of Biotherapy, West China Hospital of Sichuan University, Chengdu, 610041, China

**Keywords:** Tetrahedral DNA nanostructures, Wound treatment, Injury repair, Injury regeneration, Tissue regeneration

## Abstract

Tetrahedral DNA nanostructures (TDNs) are molecules with a pyramidal structure formed by folding four single strands of DNA based on the principle of base pairing. Although DNA has polyanionic properties, the special spatial structure of TDNs allows them to penetrate the cell membrane without the aid of transfection agents in a caveolin-dependent manner and enables them to participate in the regulation of cellular processes without obvious toxic side effects. Because of their stable spatial structure, TDNs resist the limitations imposed by nuclease activity and innate immune responses to DNA. In addition, TDNs have good editability and biocompatibility, giving them great advantages for biomedical applications. Previous studies have found that TDNs have a variety of biological properties, including promoting cell migration, proliferation and differentiation, as well as having anti-inflammatory, antioxidant, anti-infective and immune regulation capabilities. Moreover, we confirmed that TDNs can promote the regeneration and repair of skin, blood vessels, muscles and bone tissues. Based on these findings, we believe that TDNs have broad prospects for application in wound repair and regeneration. This article reviews recent progress in TDN research and its applications.

Highlights

- With advantages of unsurpassed structural stability, excellent material editability, good biocompatibility and individual endocytic pathway TDNs have been used extensively.
- Different biological effects and mechanism that TDNs play *in vitro* and *in vivo* are illustrated*.*
- Multiple clinical applications in repair and regeneration of tissues and organs are described.

## Background

The anionic property of DNA hinders its transport through cell membranes [1,2]. Meanwhile, DNA nanomaterials differ from native DNA macromolecules because they are characterized by structural stability, regulation of biological behavior and the ability to pass through cell membranes [3]. Tetrahedral DNA nanostructures (TDNs) are molecules with a pyramidal structure formed by folding four single strands of DNA based on the principle of base pairing [4]. Because of their structure, TDNs have unique advantages [5–11]. For example, adjusting their orientation can minimize charge repulsion, leading to charge redistribution. This process helps TDNs to be successfully absorbed by cells through caveolin-mediated endocytosis and then be transported into the lysosome [12]. Because of their small size, they can be assembled with an additional nuclear locator aptamer and be specifically guided to escape target lysosomes, which is the key to their successful delivery [13,14].

TDNs have excellent biocompatibility, programmability, higher density and better serum and structural stability than native DNA [11,13]. They also have been shown to have nuclease resistance [15]. Tetrahedral structures constructed by DNA origami technology can pass through the cell membrane through endocytosis and carry out their functions without obvious toxic side effects. In addition, based on its double-helix structure and base complementary pairing [16], and the use of enzymes such as DNA polymerase, DNA ligase and restriction endonucleases, DNA can be edited, folded and assembled into complex nanomaterials of different shapes and sizes [17–21].

**Figure 1.**
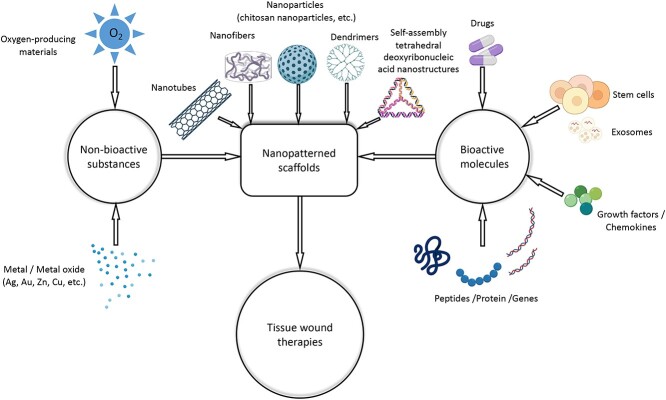
Schematic representation of composites used for wound repair and tissue regeneration

Tissue wound repair and regeneration are complex and highly coordinated processes that depend on individual health conditions and other physiological factors [22,23]. Various strategies have been developed and implemented for wound healing in different tissues based on the progress of tissue engineering [24]. These strategies relate to all kinds of bioactive molecules (such as genes, proteins, peptides, stem cells, drugs and growth factors) and non-bioactive substances (such as metal ions, oxygen-producing materials etc.) (Figure 1). A combination of nanomaterials and these substances can regulate cell proliferation and migration, promote cell differentiation and angiogenesis, inhibit inflammatory and oxidative stress responses and create a suitable healing physiological environment [25–27]. TDNs, as multifunctional nanomaterials, can directly regulate cell biological activities and deliver bioactive/non-bioactive substances to targeted tissues/organs to facilitate wound recovery [28,29].

The development of DNA nanomaterials has undergone several stages [5,6,30]. DNA nanomaterials have attracted the attention of an increasing number of researchers [5,31–39] and are extensively used in many biomedical research fields [1,40–43]. As a versatile DNA material, TDNs have been shown to fulfill multiple functions in biotherapy, drug delivery, molecular diagnostics, biological imaging and multiplexed sensing applications [29,44,45]. In particular, TDNs have been widely applied in wound repair and regeneration engineering, and rapid progress has been made in their application [28,29,46–48]. This review summarizes the progress of TDN research in wound repair and regeneration to promote further research in the field of tissue engineering [49,50].

## Review

### Synthesis and structure of TDNs

A TDN is composed of four equidistant single-stranded DNA molecules [5], with each single-stranded DNA molecule containing three modules that can hybridize with the other three strands by complementary base pairing. The rigid triangles of each DNA strand form one side of the tetrahedral structure with two oligonucleotide ends at the apex that are connected by covalent binding [51]. At the same time, each side of the TDN is separated by several unhybridized nucleotides, providing sufficient flexibility for bending [52].

**Figure 2.**
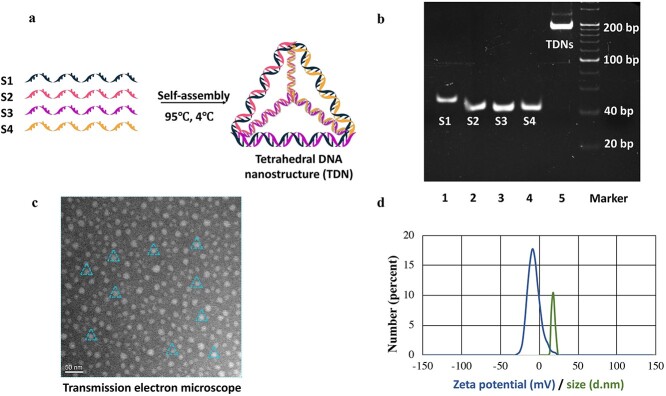
Successful synthesis and characterization of tetrahedral DNA nanostructures (TDNs). (**a**) Schematic diagram of TDNs. (**b**) Confirmation of the successful synthesis of TDNs by 8% polyacrylamide gel electrophoresis. Lanes 1–5: S1, S2, S3, S4 and TDNs, showing the successful synthesis of TDNs. (**c**) Transmission electron microscope images of TDNs. (**d**) Zeta potential graph and typical size distribution graph of TDNs

The specific method for TDN synthesis involves mixing four single-stranded DNAs in equimolar amounts in TM buffer (50 nM MgCl_2_, 10 nM HCl, pH = 8.0), heating to 95°C for 10 min and then cooling to 4°C for 30 min (Figure 2a) [46,53]. To verify the synthesis of self-assembled TDNs, we checked the molecular weights of the TDNs and the four single-stranded DNAs by 8% polyacrylamide gel electrophoresis (Figure 2b). TDN synthesis was also verified by observing their morphology and measuring their molecular weight and surface potential. Transmission electron microscopy was used to confirm the successful synthesis of the TDNs (Figure 2c). The diameter of the TDNs was ~15 nm by dynamic light scattering and the zeta potential of the TDNs was ~−6 mV (Figure 2d).

### Biological characteristics of TDNs

#### TDNs can promote cell proliferation and migration and inhibit apoptosis

In 2016, TDNs were found to promote cell proliferation in mouse fibroblast-L929 [35]; 1 year later, we found that they could also promote the migration of chondrocytes [54]. Then, we gradually started to explore the various biological effects of TDNs on cells. Over the course of several years, our research team found that TDNs could regulate PI3K/AKT/mTOR, Wnt/β-catenin, Notch and other signaling pathways [55–57]. Specifically, TDN can inhibit apoptosis and promote cell proliferation and migration by regulating the expression of cyclin-dependent kinase like-1 [35,51,58] and the DNA methylation of Dlg3 gene promoter [58], relieving the inhibitory effect of DKK-1 on the Wnt/β-catenin signaling pathway and promoting cell proliferation [51] or the expression of caspase-3, β-catenin, cyclin D1, Rho A, ROCK2 and vinculin [54,55]. Moreover, TDN can enhance the osteogenic differentiation ability of adipose mesenchymal stem cells, inhibit the apoptosis of chondrocytes and promote the regeneration and repair of muscle and other tissues, as well as accelerate cellular migration in damaged cartilage, periodontal bone tissue, corneal epithelium and nerve tissues [54,59–61] (Table 1). Thus, TDNs have demonstrated broad application prospects in the field of regenerative medicine.

**Table 1:** Related molecular mechanisms of tetrahedral DNA nanostructures treatment effects

**Biological activity**	**Organ/tissue**	**Related molecular mechanisms**	**Reference**
Cell proliferation	Skin scar	Promoting cell mitosis and causing it to proceed from the S phase to the G2/M phase.	[53,60,62,63]
	Skeletal muscle	Activating Wnt/β-catenin signaling pathway.	
	Corneal epithelium	Upregulation of the phosphorylation of ERK1/2 and p38.	
	Neural stem cell	Activating the Wnt/β-catenin pathway.	
Cell differentiation	Skeletal muscle	Maintaining the protein expression level of PAX7.	[43,60,63,64]
	Neural stem cell	Inhibiting the Notch signaling pathway.	
	Bone	Activating the Wnt/β-catenin signaling pathway. Upregulating the expression of c-Fos and NFATc1	
	Cementum or alveolar bone	Increasing the protein expression of osteogenic factors OPN and RUNX2.	
Cell migration	Corneal epithelium	Upregulation of the phosphorylation of ERK1/2 and p38.	[61,62,65]
	Articular cartilage	Promoting the expression of RHOA/ROCK2 and vinculin.	
	Bone	Downregulating lncRNA XLOC 010623 and activating RHOA/ROCK2 signaling pathway.	
	Neural stem cell	Activating RHOA/ROCK2 signaling pathway.	
Angiogenesis	Blood vessel	Notch signaling, JAK/STAT signaling and Akt/Nrf2/HO-1 pathway. Activation of the Notch signaling pathway.	[66–68]
	Bone	Regulating the phosphorylation of STATs, enhancing the expressions of angiogenesis-related growth factors (VEGFA/B, IGF, HIFα, TGFβ1, PDGF).	
Anti-apoptosis and anti-oxidative stress	Diabetic oral mucosa	Activating Akt/Nrf2/HO-1 signaling pathway.	[43,63,69–72]
	Periodontium	Increasing the intensity of Bcl-2. Reducing Bax and caspase-3 gene expression and protein intensity. Activating the Akt/Nrf-2 signaling pathway to exert the antioxidative effects.	
	Parkinson’s disease	AKT/PI3K and mitochondrial apoptotic pathways.	
	Alzheimer’s disease	Activating ERK1/2 phosphorylation to inhibit Aβ-induced apoptosis.	
	Heart muscle	Activating the Akt/Nrf2 signaling pathway.	
	Neural stem cell	Inhibit cytotoxicity caused by the abnormal deposition of Aβ and caspase 3 expression.	
Anti-inflammation	Skin scar	Activating the AKT-signaling pathway.	[39,43,53]
	Periodontium	Decreasing the protein level of ERK, JNK and P38 to inhibit MAPK/ERK signaling pathway.	
	Articular cartilage	Downregulating the gene expression of MMPs and TNF-α. Suppressing the activation of NF-κB p65 and inhibited the degradation of IκBα.	
	Bone	Regulating the phosphorylation of STATs, promoting the M2 phenotype polarization of macrophages and secreting IL-10, TGF-β and other anti-inflammatory factors.	
Autophagy	Articular cartilage	PI3K/Akt/mTOR signaling pathway.	[39,60,73]
	Skeletal muscle	Enhancing the protein expression of LC3 and Beclin1	
	Parkinson’s disease	PI3K/Akt/mTOR signaling pathway.	

*S phase* synthesis phase, *G2 phase* growth 2 phase, *M phase* mitosis phase, *ERK* extracellular signal-regulated kinase, *PAX7* paired box protein Pax-7, *JNK* c-Jun N-terminal kinase, *NFATc1* nuclear factor of activated T-cells cytoplasmic 1, *OPN* osteopontin, *RUNX2* runt-related transcription factor 2, *RHOA* Ras homolog family member A, *ROCK2* Rho associated coiled-coil containing protein kinase 2, *lncRNA* long non-coding RNA, *JAK* Janus kinase, *STAT* signal transducer and activator of transcription, *Akt* protein kinase B, *Nrf2* nuclear factor erythroid 2-related factor 2, *HO-1* heme oxygenase-1, *Bcl-2* B-cell lymphoma 2, *BAX* Bcl-2-associated X-protein, *VEGFA/B* vascular endothelial growth factor A/B, *IGF* insulin-like growth factors, *HIFα* hypoxia-inducible factor α, *TGFβ1* transforming growth factor beta 1, *PDGF* platelet-derived growth factor, *Aβ* amyloid beta, *PI3K* phosphoinositide 3-kinase, *MAPK* mitogen-activated protein kinase, *MMPs* matrix metalloproteinases, *NF-κB* nuclear factor kappa-light-chain-enhancer of activated B cells, *IκBα* nuclear factor of kappa light polypeptide gene enhancer in B-cells inhibitor alpha, *M2* M2-like macrophages, *IL-10* interleukin 10, *TGF-β* transforming growth factor beta, *LC3* microtubule-associated proteins 1A/1B light chain 3B, *mTOR* mammalian target of rapamycin

**Table 2:** Biological activities of tetrahedral DNA nanostructures in wound repair and tissue regeneration

**Tissues/** **organs**	**Wound type**	**Model**		**Biological activity**	**Reference**
		***In vitro***	***In vivo***		
Corneal epithelium	Corneal alkali burn model	Human corneal epithelial cells (HCECs)	Rabbits	Promoting proliferation and immigration of HCECs. Maintaining corneal transparency and accelerating re-epithelialization.	[62]
Skin	Skin wound	Human skin fibroblast (HSF) cell lines/human epidermal keratinocyte (HaCaT) cell lines	Sprague-Dawley (SD) rats	Promoting cellular proliferation by modulating cell cycle and increasing cellular migration. Increasing the secretion of growth factors in HSF cells. Relieving inflammatory reactions in HaCaT cells. Reducing fibrosis and inflammation in the scar area *in vivo*. Accelerating cutaneous wound closure and decreased scar formation *in vivo*.	[53]
Mucosa	Diabetic oral mucosa traumatic wound model	Not applicable (N/A)	Wistar rats	Facilitating diabetic wound healing by accelerating epithelialization, vascularization, collagen deposition and alignment.	[69]
Periodontium	Ligature-induced periodontitis model	Human periodontal ligament stem cells (PDLSCs)	SD rats	Promoting migration and osteogenic differentiation of PDLSCs. Antioxidant and anti-inflammatory on PDLSCs. Inhibiting inflammatory response and the destruction of periodontal tissue *in vivo*.	[43]
Blood vessel	Wound vascularization	Human umbilical endothelial cells (HUVECs)	Matrigel plug in BALB/c nude mice	Promoting the proliferation and migration of HUVECs. Regulating the expression level of angiogenic growth factor. Stimulating formation of endothelial tubes and endothelial sprouting. Facilitating microvessel formation *in vivo*.	[67]
	Bisphosphonates inhibited angiogenesis	Human umbilical endothelial cells	Wistar rats	Reversing the proliferation inhibition effects of zoledronic acid on HUVECs. Promoting migration and angiogenesis ability of HUVECs. Regulating angiogenesis and macrophage polarization *in vivo*.	[68]
Skeletal muscle	Acute muscle injury	C2C12 cells	C57BL/6 mice	Promoting C2C12 cell proliferation. Enhancing autophagy levels in C2C12 cells. Maintaining the stemness of myoblasts during skeletal muscle regeneration. Accelerating the healing process of acutely injured muscle *in vivo*. Increasing the number of myoblasts *in vivo*.	[60]
Heart muscle	Myocardial ischemia–reperfusion injury	H9c2 cells	N/A	Inhibiting simulated ischemia–reperfusion injury (SIR) cytotoxicity of H9c2 cells. Reducing the production of ROS to depress oxidative damage. Regulating the expression of apoptosis-related genes and proteins to inhibit cell apoptosis.	[72]
Bone	Bony defect	Rat adipose-derived stem cells (ADSCs)	N/A	Stimulating the osteogenic differentiation and proliferation of ADSCs. Enhancing the migration of ADSCs.	[64,65]
	Tooth extraction-induced bisphosphonate-associated osteonecrosis of the jaw (BRONJ)	Raw 264.7 cells	Wistar rats	Promoting cell proliferation and migration. Inhibiting zoledronic acid-induced cytotoxicity *in vitro*. Promoting maturation and differentiation of osteoclasts. Stimulating macrophage polarization to M2 phenotype to possess anti-inflammatory and antioxidative effects. Inhibiting bone destruction and promoting the healing of the tooth socket after extraction *in vivo*.	[68,81]
	Periodontitis-induced cementum or alveolar bone resorption	N/A	SD rats	Attenuating inflammatory cells infiltration. Downregulating the expression of proinflammatory factors and inhibiting osteoclast genesis. Inhibiting the absorption of cementum and alveolar bone.	[43]
Articular cartilage	Inflammatory chondrocytes	Rats’ knee-joint chondrocytes	N/A	Facilitating the proliferation and migration of chondrocytes.	[54]
	Osteoarthritis	SD rat articular chondrocytes	Wistar rats	Inhibiting cell apoptosis, increasing chondrogenic marker expression. Alleviating inflammation by suppressing the expression of inflammatory mediators. Promoting chondrocyte regeneration *in vitro* and *in vivo*.	[39]
Nerve tissue	Nerve tissue regeneration	Mouse neuroectodermal (NE-4C) stem cells	N/A	Promoting the proliferation and neuronal differentiation of the stem cells. Facilitating neural stem cell migration.	[61,63]
	Neurotoxin-1-methyl-4-phenyl-1,2,3,6-tetrahydropyridine (MPTP)-induced Parkinson’s disease (PD) cell model	Rat pheochromocytoma cells line (PC12 cells)	N/A	Inhibiting the cytotoxicity and apoptosis caused by MPTP. Reducing the abnormal accumulation of α-synuclein.	[70]
	Alzheimer’s disease (AD) model	PC12 cells	SD rats	Increasing cell viability and reducing AD-induced apoptosis in PC12 cells. Improving learning and memory in an AD rat model. Reducing Aβ1–40 deposition and apoptosis in the rat hippocampus.	[71,82]

#### TDNs have anti-inflammatory and antioxidant effects

Our research has also found that TDNs can significantly inhibit the expression of pro-inflammatory factors such as interleukin-1β (IL-1β), IL-6 and tumor necrosis factor α (TNF-α) [74]. TDNs can upregulate the expression levels of antioxidant enzymes, thereby inhibiting cell apoptosis. TDNs have also been found to have an anti-infective effect, which may be related to their reactive oxygen species (ROS) clearance function [53,69]. In previous studies, we found that TDNs could inhibit tissue inflammation by activating Akt/Nrf2/hemeoxygenase-1 (HO-1) or other signaling pathways [53,69] and regulating the expression levels of BCL2, BAX, caspase-3, Nrf2, beclin1 and β-catenin [55,75]. In addition to reducing lipopolysaccharide-induced ROS, TDNs can reduce oxidative damage and regulate the expression of apoptosis-related proteins (Table 1). All these pathophysiological changes show anti-inflammatory and antioxidant effects [72], which contribute to protecting the heart muscle from myocardial ischemia–reperfusion injury (MIRI) to help treat fibrotic diseases [76].

#### TDNs have anti-infective and immune regulation activities

TDNs have been found to have anti-infective effects and regulate innate and adaptive immunity [42,77] (Table 1). For example, in tissue wounds, biofilm formation is an important factor that affects tissue repair and leads to chronic tissue wounds [78,79]. Biofilms may become more adherent and resistant to antibiotics due to bacterial and extracellular polysaccharides (EPS). TDNs can help inhibit the synthesis of EPS and reduce the thickness of biofilms, showing their potential to treat chronic infections caused by biofilms [60,61]. Furthermore, they can inhibit the activation of Th1 and Th17 cells, promote the activation of Th2 and Treg cells and regulate the expression levels of some targeted genes. TDNs can ultimately be used to improve insulin resistance [42, 81] and treat chronic infections caused by biofilms [37,80] or multiple bacterial infections [38].

Therefore, because of their 3D structure and cell membrane permeability, especially their effects in promoting cell proliferation, cell migration and anti-apoptotic, anti-inflammatory, antioxidant, anti-infective and immune regulation functions (Table 1), we systematically explored the role of TDNs in tissue regenerative medicine. The following is a summary of our research results in different areas of wound repair and regeneration, which we have confirmed in recent years (Table 2).

### Applications of TDNs in repair and regeneration of multiple tissues and organs

#### TDNs can facilitate epithelial tissue wound healing and reduce scar formation

**Figure 3.**
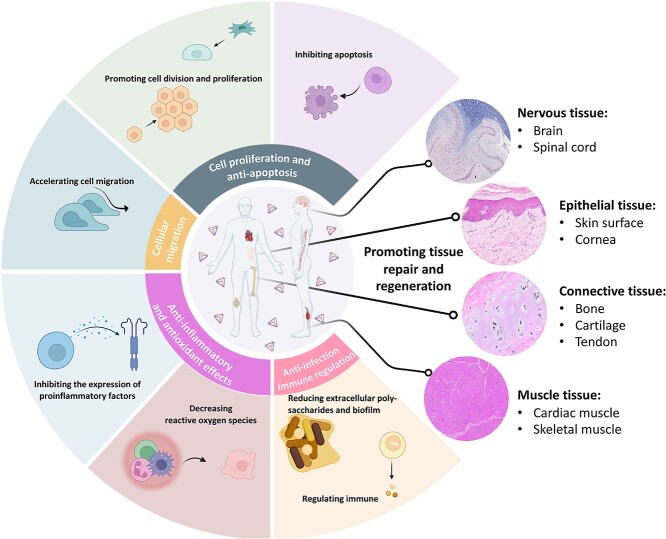
Tetrahedral DNA nanostructures play a vital role in repair and regeneration in several tissues

Wound healing involves a series of processes involving wound tissue regeneration, granulation tissue hyperplasia and skin scar tissue formation. Corneal epithelial wound healing is more complicated and includes cell proliferation, death and migration [83,84]. Poor wound healing in this area may lead to blindness [85]. In recent years, there have been many published studies related to corneal wound healing [86–89], but the development of drugs with curative effects remains limited. With the rapid development of nanotechnology, TDNs have been designed for a variety of biological functions, including cell proliferation, anti-inflammatory activity and osteogenic differentiation [4,43,53]. In view of the advantages of TDNs, we established a rabbit corneal alkali burn model and explored the effect of TDNs on the healing of corneal epithelial injuries. We found that TDNs may increase corneal transparency and wound re-epithelialization by regulating the activation of the P38 and ERK1/2 signaling pathways [62]. This shows that TDNs can play a vital role in the healing of corneal epithelial wounds. In addition, TDNs can also effectively reduce scar formation. As the human body’s first line of defense, wounding often occurs on the skin, and the healing of skin wounds involves a complex process that includes inflammation, tissue formation and tissue remodeling [90]. Owing to collagen deposition and the formation of granulation tissue during the process of wound healing, scars are usually left on the skin [90,91]. We explored the effect of TDNs on both cells *in vitro* and rat trauma models *in vivo*. We found that TDNs stimulate the production of vascular endothelial growth factor and basic fibroblast growth factor in human skin fibroblast cells. Meanwhile, TDNs regulate the inflammatory immune response by activating the AKT signaling pathway, which inhibits the secretion of TNF-α and IL-1β *in vitro*. We also found that after TDN treatment, the healing rate of skin wounds was significantly improved, fibrosis and inflammation in the scar area were significantly reduced and scar formation was ultimately reduced [53]. These results demonstrate the role of TDNs in promoting skin regeneration and their potential for accelerating tissue and organ wound healing (Figure 3) [35].

#### TDNs can promote the regeneration and repair of blood vessels

Vascularisation is critical for the survival of tissue engineering grafts. Researchers have made a series of explorations in this field, including the use of growth factors and endothelial cells to form composite scaffolding materials or microsurgery techniques to promote angiogenesis [92–94]. Nevertheless, most methods for rebuilding blood supply are not yet well organized. Our previous research found that TDNs are effective in angiogenesis. TDNs can enter endothelial cells and promote the proliferation and expression of angiogenic growth factors as well as the formation of the lumen by activating the Notch signaling pathway [66]. Another study confirmed the effect of TDNs in promoting the formation of individual blood vessels [81]. An *in vivo* Matrigel plug assay demonstrated the excellent angiogenesis-promoting effects of TDNs. Compared with the control group, TDNs could notably induce neo-vessel formation 7 days after a mixture of human umbilical vein endothelial cells and Matrigel was injected into the right ventral side of nude mice for 7 days [67]. In addition, we found that TDNs can reduce the expression level of ROS associated with oxidative damage and inflammation in diabetes, and promote wound healing by promoting vascularisation and epithelialisation [69]. These findings provide evidence for the application of TDNs in tissue engineering vascularization.

#### TDNs can promote muscle regeneration and repair

##### TDNs can promote muscle regeneration

Muscles may deteriorate after injury, which affects their physical appearance and motor function [60]. Muscle regeneration is an important branch in tissue regeneration, but satisfactory methods for muscle regeneration are still lacking, especially for senescent cells with limited muscle repair ability. In recent years, tissue engineering technology has gradually become popular for repairing muscle tissue injuries [95,96]. Autophagy, in particular, has great potential in tissue and muscle regeneration, as upregulating autophagy could help maintain muscle cell homeostasis and self-renewal, thus reducing aplastic diseases [97,98].

As an editable and stable nanomaterial [1,35], TDNs were previously found to promote cell growth, be biocompatible with proliferation and have anti-inflammatory and pro-migratory effects [28,58,74]. Therefore, we used TDNs to treat a mouse model characterized by acute muscle injury and explored the function of TDNs in the proliferation and regeneration of skeletal muscle. Our results showed that TDNs can regulate the Wnt/β-catenin signaling pathway and autophagy of myoblasts, promote the proliferation of skeletal muscle cells and maintain the properties of stem cells *in vivo* [60]. This indicates that TDNs have good prospects for skeletal muscle regeneration (Figure 3).

##### TDNs have a protective effect on heart muscle

Acute myocardial infarction is the most serious ischemic heart disease of the coronary arteries, and timely reperfusion can help reverse myocardial ischemia [99–101]. Nevertheless, MIRI caused by reperfusion or ROS produced by apoptosis has serious impacts on cardiomyocytes [102,103]. At the beginning of reperfusion, enormous amounts of ROS are produced, followed by subsequent calcium overloading, caspase activation, upregulation of cytokines and peroxidation of DNA and proteins, which induces further cell apoptosis [103]. Previous studies have suggested that by increasing HO-1, TDNs could reduce ROS produced by macrophages and inhibit neuronal apoptosis [74]. In our recent study, we found that treatment with TDNs significantly decreased the levels of lactate dehydrogenase, which is a vital biomarker of cell death. In addition, by activating the Akt/Nrf2 signaling pathway, TDNs could significantly reduce oxidative damage, apoptosis and the overexpression of ROS-induced apoptosis proteins [72]. These findings suggest that TDNs can protect against myocardial injury in MIRI.

#### TDNs can promote the repair and regeneration of bone tissue

##### TDNs can promote the migration and osteogenic differentiation of adipose-derived mesenchymal stem cells

Bone tissue defects are common in clinical practice and are usually caused by trauma and infection. Reconstruction of the defective bone tissue remains a challenge for surgeons. Adipose-derived mesenchymal stem cells (ADSCs) have attracted attention in the clinical application and development of orthopedic regenerative medicine, especially because they are rich in resources, easy to isolate and cause minimal trauma. They are found to be an excellent source of bone marrow mesenchymal stem cells for clinical application in bone tissue regeneration [104–107]. To date, they are widely used in bone defect repair and reconstruction. Unfortunately, the osteogenic differentiation ability of TDNs for large bone defects is still insufficient [108,109]. TDNs can be absorbed by cells without other adjuvants and transported to endocytosis through vesicle protein mediation.

Accumulating evidence has shown that TDNs are important regulators of cell proliferation [12,15]. In a previous study, we found that TDNs can affect ADSCs by regulating the RHOA/ROCK2 signaling pathway [65]. Furthermore, we found that TDNs could also enhance the osteogenic differentiation of ADSCs. In an attempt to explore how TDNs affect the proliferation and osteogenic differentiation of ADSCs [110], we found that TDNs can activate the Wnt/β-catenin signaling pathway, improve alkaline phosphatase activity and promote calcium deposition. This shows that TDNs may be a potential repair method for bone tissue engineering research.

##### TDN can influence DNA methylation in stem cells

In addition to promoting the proliferation, migration, differentiation and osteogenesis of ADSCs, other effects of TDNs at the epigenetic level have also aroused great interest. We further explored the changes in stem cell DNA methylation and gene expression after ADSCs were treated with TDNs. We found that TDNs could also regulate DNA methylation levels, thereby promoting ADSC proliferation and inhibiting apoptosis [58]. Our research provides a solid foundation for the application of TDNs and provides a deeper understanding of the proliferation and anti-apoptotic capabilities of TDNs.

##### TDNs can promote the proliferation, migration and autophagy of chondrocytes

Osteoarthritis is a common degenerative pathological process of articular cartilage that is accompanied by excessive chondrocyte apoptosis. Cartilage tissue is a non-vascular connective tissue covering the articular surface and plays an important role in the maintenance of normal biological functions. However, owing to their poor self-repair ability, it is of great importance to use cells, biological materials and other stimuli to jointly regulate the regeneration of cartilage cells, thereby promoting the repair of damaged cartilage tissue.

Based on the anti-apoptotic and antioxidant effects of TDNs in a variety of diseases, we further explored their effect on chondrocytes. We found that TDNs can be internalized by chondrocytes without other auxiliary agents, and are mainly concentrated in the cytoplasm. In addition, chondrocyte autophagy is enhanced by the activation of the PI3K/AKT/mTOR signaling pathway by TDNs [59]. Furthermore, we found that TDNs can activate the RHOA/ROCK2 signaling pathway, thus influencing the culture environment and migration of chondrocytes [54]. Finally, by promoting autophagy and inhibiting the Wnt/β-catenin signaling pathway, TDNs inhibit chondrocyte apoptosis and oxidative stress and promote cartilage tissue regeneration [51,55]. In addition, we studied the effect of TDNs on jaw and periodontal tissues. TDNs are effective in repairing jaw necrosis and promoting proliferation and osteogenesis of dental pulp stem cells [68,81] as well as the proliferation and differentiation of periodontal ligament stem cells (PDLSCs) [56,57] (Figure 3).

##### TDNs can reduce alveolar bone absorption and protect periodontal tissue

As a common inflammatory oral disease, periodontitis significantly affects the quality of life of patients [111]. A previous study showed that plaque is the main cause of periodontitis. This inflammatory change in bone tissue can lead to resorption of periodontal bone and may even ultimately result in tooth loss if not properly treated [112]. Moreover, the inflammatory microenvironment has a negative influence on periodontal regeneration, osteogenic differentiation and the migration of PDLSCs. Thus, alleviating the inflammatory process and facilitating the regeneration of periodontal tissue and alveolar bone are important treatment targets for periodontitis.

*In vitro*, we found that the MAPK/ERK signaling pathway, which contributes to PDLSC inflammation, was inhibited by TDN treatment. Compared with the group treated with lipopolysaccharides (LPS) alone, the group treated with both LPS and TDNs showed elevated protein levels of ERK, JNK and P38. Meanwhile, even under inflammatory conditions, TDNs can promote the osteogenic differentiation of PDLSCs. The gene expression of *ALP* and *RUNX2* in PDLSCs was enhanced and the synthesis of osteogenic factors such as OPN and RUNX2 was increased, suggesting that TDNs can improve osteogenic capacity. An animal model of periodontitis was established using the second molar of Sprague-Dawley (SD) rats. Except for the influence of height or density, alveolar bone tissue in the inflammatory model remained almost the same as in the control group after treatment. The number of osteoclasts, the level of pro-inflammatory factors IL-1β and IL-6 and the infiltration of inflammatory cells all decreased significantly in the periodontium after TDN treatment. The alveolar bone was greatly repaired to its normal form by TDNs [43].

These studies suggest that TDNs have great potential for bone tissue repair and regeneration.

#### TDNs can promote the regeneration and repair of nerve tissue

##### TDNs regulate the migration, proliferation and differentiation of neural stem cells

Stem cell therapy has been considered a promising method for repairing damaged nerve tissues [113–117]. However, because neural stem cells (NSCs) cannot effectively proliferate or differentiate, it is imperative to explore ways to effectively improve their proliferation and differentiation. For this reason, we explored the effects of TDNs on cell self-renewal and differentiation and found that TDNs had a significant stimulatory effect on the proliferation of NSCs and the differentiation of neurons [63].

Despite these findings, it is still difficult to determine the effect of TDNs in promoting NSC migration, which is a complex biological process [118–120]. The key to the repair of neural cells is the migration and differentiation of NSCs into the damaged nerve tissue. Unfortunately, the ability of NSCs to proliferate, migrate and differentiate is poor, and injured nerve tissue is difficult to repair or regenerate [121–123]. Therefore, we explored the effect of TDNs on the migration of neuroectoderm (NE-4C) stem cells and revealed the underlying mechanism. We found that TDNs can promote the migration of NSCs by activating the RHOA/ROCK2 signaling pathway and thus have important potential in the regeneration and repair of neural tissues [61]. These results suggest that NSC migration has broad application prospects in nerve tissue repair and regeneration.

##### TDNs can protect and repair nerves

Alzheimer’s disease (AD) is a common neurodegenerative disease that usually manifests as progressive neurocognitive dysfunction and neuronal damage [124–126]. Clinical drugs for AD are still limited [126–128]. Based on the neuroproliferative and protective effects of TDNs, we further studied the role of TDNs in neuronal cell proliferation and apoptosis [71]. We confirmed that TDNs can effectively promote cell proliferation and inhibit apoptosis in AD model cells. We also found that TDNs can reduce Aβ deposition and help improve memory and learning abilities in a rat model of AD [82]. These studies shed new light on the field of nerve regeneration, in which TDNs may become a potential new strategy for the prevention and treatment of AD.

In addition, Parkinson’s disease (PD), a neurodegenerative disease with an incidence rate second to AD, has attracted our attention [129]. The main clinical symptom of PD presents as a series of progressive dyskinesia. The current drugs used to treat PD are far from effective and have obvious side effects. Therefore, it is vital to explore efficient drugs for PD with fewer side effects. TDNs can reduce ROS levels and abnormal accumulation of α-synuclein, and inhibit cell apoptosis. This suggests that TDNs may be a new drug candidate for the treatment and prevention of PD [70] (Figure 3).

### Problems and prospects

TDNs have multiple biological efficacies, unsurpassed structural stability, excellent material editability, good biocompatibility and individual endocytic pathways. Because of these traits, TDNs have been used extensively in disease treatment research, especially in the repair and regeneration of tissues and organs. New opportunities and challenges have been introduced in this promising field of nanomedicine.

However, the development of TDN materials faces some obstacles. First, the high cost of synthesis may limit mass production and large-scale clinical application of TDNs in tissue repair and regeneration. To solve this problem, the mirror structure of TDNs is obtained by DNA origami technology, which maintains the TDN structure to guarantee long-lasting efficacy, stronger serum stability and longer physical retention times. Second, most studies were conducted *in vitro* or in mice, rats and rabbits, which differ in histomorphology and pathophysiological mechanisms from humans. These differences make it necessary to conduct similar studies on large animals, such as pigs or primates, to further verify the clinical effects and application of TDNs in tissue repair and regeneration. Third, long-term cytotoxicity is a concern. Oligonucleotides are biodegradable and biocompatible; however, their properties may change when DNA is designed as nanostructures. There are unknown risks and benefits, especially in terms of the long-term cytotoxicity and biological availability of TDNs under physiological conditions. Therefore, dynamic and longitudinal follow-up and in-depth research should be conducted. At the same time, the use of DNA origami technology can promote diverse designs and modifications to the structure and function of TDNs, which may remedy these limitations. Hopefully, these challenges can be addressed through multidisciplinary collaboration among clinical doctors, biologists and material engineers.

## Conclusions

In summary, the special spatial structure and small size of TDNs enable them to enter cells without the aid of transfection agents and participate in mediating cellular functions. TDNs can promote cell migration, proliferation and differentiation, and they possess anti-inflammatory, antioxidant, anti-infective and immune regulation capabilities. These properties can promote skin wound healing, reduce scar formation and promote the regeneration and repair of vascular, musculoskeletal and nerve tissues. We believe that TDNs have great potential for application in wound repair and regeneration. Further research is urgently required in this promising field.

## Authors’ contributions

YD, WC, XY, YL, XM and XC wrote the manuscript. All authors have read and approved the final manuscript.

## Funding

This research was supported by the National Key R&D Program of China (No. 2019YFA0110600), National Natural Science Foundation of China (Nos. 82001432, 81970916), China Postdoctoral Science Foundation (Nos. 2020TQ0213, 2020 M683319) and West China Hospital Postdoctoral Science Foundation (No. 2020HXBH104).

## Conflict of interest

None declared.

## Abbreviations

AD: Alzheimer’s disease; ADSCs: Adipose-derived mesenchymal stem cells; EPS: Extracellular polysaccharides; HO-1: Hemeoxygenase-1; IL-1*β*: Interleukin-1*β*; LPS: Lipopolysaccharides; MIRI: Myocardial ischemia–reperfusion injury; NSCs: Neural stem cells; PD: Parkinson’s disease; PDLSCs: Periodontal ligament stem cells; ROS: Reactive oxygen species; TDNs: Tetrahedral deoxyribonucleic acid nanostructures; TNF-*α*: Tumor necrosis factor *α*.
